# The Neuropeptide Systems and their Potential Role in the Treatment of Mammalian Retinal Ischemia: A Developing Story

**DOI:** 10.2174/157015913804999423

**Published:** 2013-01

**Authors:** D Cervia, G Casini

**Affiliations:** Department for Innovation in Biological, Agro-food and Forest systems (DIBAF), University of Tuscia, Viterbo, Italy

**Keywords:** Angiotensin, glutamate release, neuronal death, PACAP, peptide receptors, opioid peptides, somatostatin.

## Abstract

The multiplicity of peptidergic receptors and of the transduction pathways they activate offers the possibility of important advances in the development of specific drugs for clinical treatment of central nervous system disorders. Among them, retinal ischemia is a common clinical entity and, due to relatively ineffective treatment, remains a common cause of visual impairment and blindness. Ischemia is a primary cause of neuronal death, and it can be considered as a sort of final common pathway in retinal diseases leading to irreversible morphological damage and vision loss. Neuropeptides and their receptors are widely expressed in mammalian retinas, where they exert multifaceted functions both during development and in the mature animal. In particular, in recent years somatostatin and pituitary adenylate cyclase activating peptide have been reported to be highly protective against retinal cell death caused by ischemia, while data on opioid peptides, angiotensin II, and other peptides have also been published. This review provides a rationale for harnessing the peptidergic receptors as a potential target against retinal neuronal damages which occur during ischemic retinopathies.

## NEUROPEPTIDES AND PEPTIDERGIC RECEPTORS

Peptidergic signaling molecules are widely distributed throughout the central and peripheral nervous systems and in the peripheral organs. They act in neurocrine, paracrine, autocrine and endocrine manners and the same peptides may participate in intercellular communications through different modalities. Neuropeptides play a crucial role in the normal function of the central nervous system (CNS) [1]. They may be costored with other neuropeptides or, alternatively, may coexist with other messenger molecules, as for instance “classical” neurotransmitters, in different cellular compartments [2]. Functionally, specific permutations of transmitters may comprise a “chemical code” for neurons subserving specific actions. Costored peptides and classical transmitters are released together at all terminals of a given neuron likely acting together in determining the response of target cells. It is a general rule that when a peptide and a classical transmitter coexist, the first mediates long-lasting responses and the latter short-term synaptic events in the target cells.

Independent of the modality, peptides act through specific receptors that are generally located on the plasma membrane. Peptidergic receptors belong to the superfamily of heterotrimeric G-protein coupled receptors (GPCRs) which are characterized by the presence of 7 transmembrane domains. In many cases, there are several receptors for one peptide. Also, many naturally occurring peptides exhibit a high degree of promiscuity across GPCRs. The physiological significance of this phenomenon and the degree to which it occurs is not well characterized but it does not seem an exception in the nervous system. 

Functionally, there is a general agreement that neuropeptidergic receptors are coupled to multiple components of transduction pathways [3]. The diversity of the transduction pathways reflects the pleiotropic actions of peptides. The prevailing mechanisms in any given cell depends on many factors, including receptor distribution and the signaling elements expressed by the cell. In addition, at the cellular level, many mechanisms are involved in the amplification effects which occur in the signaling cascade. In particular, post-translational modification of receptors, different expression of factors interacting with G-proteins, and differences in G-protein repertoire and signaling isoenzymes may affect function in a process which is generally referred to as receptor trafficking [3-6]. On the other hand, it is assumed that multiple ligand-specific receptor conformations exist which may differentially couple to the intracellular pathways within the signal transduction network [7]. Indeed, peptidergic receptor reserve, coupling efficiency and probably receptor trafficking may not only depend on transduction mechanisms, but also on the nature of the ligand and the receptor at play. 

The multiplicity of peptidergic receptors and of the transduction pathways they activate offers the possibility of important advances in the development of specific drugs for clinical treatment of CNS disorders [1, 3, 8]. Peptides display high speciﬁcity for their receptors, which are activated by minimal amounts of peptide, and have minimal cross-reactivity. Different from other small molecule therapeutics, peptides do not accumulate in tissues and they are efficiently metabolized by endogenous enzymes. In addition, they have only limited potential for drug-drug interactions and are free of important toxicological complications. However, the introduction of peptide-based drugs into clinical practice is hampered by a number of technical issues that are still to be solved, as for instance *in vivo* stability, route of administration and blood brain barrier penetration. 

## ISCHEMIC RETINA

Ischemia can be defined as inadequate blood supply to a local area due to impairment of the blood vessels in that area. Ischemia may also mean that blood flow is blocked, or that oxygen saturation in the blood flowing to the area is extremely low. An ischemic state often results in the shutdown of the area or in significant damage to the area. Both oxygen and glucose delivery are impaired during ischemia and toxic metabolites cannot be removed. 

Ischemia should be distinguished from simple anoxia (a complete lack of oxygen) or hypoxia (a reduction in oxygen) [9]. Hypoxia means a reduction of oxygen availability or utilization, and it may develop as a consequence of reduced oxygen supply, reduced ambient pO_2_, low hemoglobin or reduced tissue utilization caused by impairments in the mitochondrial cytochrome enzymes. In contrast, ischemia consists in a reduction of blood supply leading not only to decreased oxygen supply, but also to decreased nutrient delivery and limited or no removal of damaging cellular metabolites. Ischemia usually coexists with hypoxia, although ischemia and hypoxia are characterized by distinct patterns of injury. Ischemia always has a component of hypoxia/anoxia, but hypoxia/anoxia does not imply ischemia. 

The mammalian retina obtains a limited amount of energy directly from the vitreous humor. Therefore, the retina can survive longer than expected if the retinal blood supply is completely blocked. However the retina is still one of the most metabolically demanding tissues in the body and it is highly vulnerable to diseases that affect the interplay between the neural retina and the vasculature that nourishes it. Generally, retinal diseases fall within the broad group of hypoxic ischemic disorders of neural tissue. When the retinal circulation becomes insufficient to meet the energy requirements of the retina, the retina suffers an ischemic damage. 

Retinal ischemia is a common clinical entity and, due to relatively ineffective treatment, remains a common cause of visual impairment and blindness [9-11]. Indeed, ischemia in the retina and optic nerve is assumed to be involved in the pathogenesis of major vision-threatening diseases, such as age-related macular degeneration, diabetic retinopathy and glaucoma. However, despite evidence from a substantial number of clinical and experimental studies, the role of retinal ischemia in these diseases is not understood in detail [12]. It should be noted that the cause of the symptoms in various retinal ischemic diseases is a mixture of hypoxia/anoxia rather than complete ischemia suggesting that hypoxia occurs in all “retinal ischemic diseases” [13]. 

**Fig. (1):**
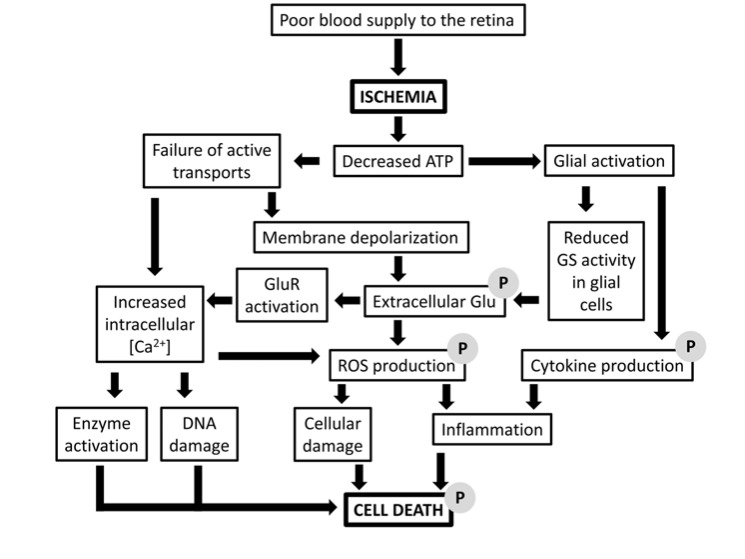
Simplified diagram showing the main processes activated in the retina by an ischemic condition and leading to neuronal death mostly by apoptosis. Effects of neuropeptides have been observed at the sites indicated by “P”. Abbreviations: Glu, glutamate; GluR, glutamate receptors; GS, glutamine synthetase; ROS, reactive oxygen species.

Generally, ischemia leads to impaired homeostatic responses which, in turn, provoke tissue injury due to cell loss by apoptosis [9-10, 13]. Ischemia is a primary cause of neuronal death in the retina, and it can be considered as a sort of final common pathway in retinal diseases leading to irreversible morphological damage and vision loss. As reviewed previously [9], although transient loss of both glucose and oxygen is not immediately lethal, the prolonged deprivation of these substrates leads to depletion of ATP stores and to retinal damage. The death of retinal neurons is the final outcome deriving from an extremely complex cascade of biochemical responses initiated by energy failure. The main factors involved in ischemia-induced retinal degeneration are thought to be excitatory neurotransmitter release (i.e., glutamate), glial dysfunction, Ca^2+^ overload, formation of reactive oxygen species and free radicals (oxidative stress), and release of potentially toxic mediators by activated inflammatory cells (Fig. **1**). These events finally lead to death (mostly by apoptosis) of certain cell populations or the entire retina depending on the strength and duration of the ischemic event. 

Besides the common clinical implications of understanding retinal ischemia, such a model is also considered a suitable and reliable experimental setting to study (neuro) apoptotic mechanisms as well as for predicting (neuro) cytotoxicity/(neuro) cytoprotection mechanisms in the nervous system. Indeed, as the retina can be studied non-invasively, the investigations on retinal ischemia may lead to a better understanding of cerebral ischemia [11]. Of importance, ischemia is also the driving force for new vessel formation in the retina. Retinal neovascularization is a major cause of visual impairment. It is characterized by the development of sprouts from retinal vessels which in most cases penetrate the inner limiting membrane growing into the vitreous humor and leading to retinal detachment and blindness [14]. Retinal neovascularization is observed in ischemic retinopathies such as proliferative diabetic retinopathy, retinopathy of prematurity, central vein occlusion and branch retinal vein occlusion [15].

## NEUROPEPTIDES AS ANTI-ISCHEMIC AGENTS

Several distinct factors are involved in the degenerative events promoted in the retina by an ischemic condition. The mechanisms leading to neuronal damage and the interplay among these factors are extremely complex. In the following paragraphs, we will consider the pathophysiological role of naturally occurring molecules such as neuropeptides.

Neuropeptides and their receptors are widely expressed in mammalian retinas, where they exert multifaceted functions both during development and in the mature animal [16]. Some of these neuropeptides have also been found to play important neuroprotective actions (Fig. **1**). In particular, in recent years, somatostatin (somatotropin release inhibiting factor, SRIF) and pituitary adenylate cyclase activating peptide (PACAP) have been reported to be highly protective against retinal cell death caused by ischemia, while data on opioid peptides, angiotensin II, and other peptides have also been published.

### Somatostatin

SRIF is a tetradecapeptide that was first identified in the hypothalamus [17]. Two forms of SRIF have been recognized, SRIF-14, the form identified originally, and SRIF-28, a congener of SRIF-14 extended at the N terminus that was discovered subsequently [18]. SRIF-14 is virtually the only form in retina [19-20]. Five SRIF subtype receptors, displaying differences in their pharmacological and functional properties, have been cloned and designated sst1 through sst5 receptors [18, 21-23]. Of interest, different SRIF analogs have been developed as multiligand compounds [24, 25].

SRIF receptors have been immunohistochemically localized to different retinal cell populations, suggesting SRIF actions at multiple levels of retinal circuitry. In particular, the sst1 receptor is expressed by SRIF-containing amacrine cells [26-28] and modulates SRIF release [29] as well as SRIF retinal levels [28], indicating that it may function as an autoreceptor. Of the two sst2 receptor isoforms, sst2A receptor has been immunohistochemically localized in rat, rabbit and mouse retinas, where it is mainly expressed by amacrine and bipolar cells [26, 30-35]. The expression of sst2A receptors by bipolar cells is consistent with the observation that SRIF may control the retinal release of glutamate through the activation of sst2 receptors [36]. This is also consistent with previous results reporting a SRIF induced, sst2 receptor-mediated down-regulation of Ca^2+^ influx in dissociated rod bipolar cells [33]. The sst2B receptor isoform in the rat retina is mainly found on the membrane of photoreceptors, indicating SRIF actions in the outer retina [34]. Finally, sst4 receptor immunolabeling in mouse retinas is localized to sparse ganglion cells [35], while localization patterns of sst5 have also been reported in rat [37] and in mouse [38] retinas.

There is wide experimental evidence that SRIF or its analogues, mainly acting at sst2 receptors, may exert potent protective effects against ischemia. In studies with an *ex vivo* model of the rat retina, SRIF analogues have been reported to significantly reduce the ischemic damage suffered by retinal neurons [39]. These observations have been replicated in an *ex vivo* model of the ischemic mouse retina, where the neuroprotective effects of SRIF have been shown to be mediated by the sst2 receptor. Indeed, these studies showed that an increased expression of functional sst2 receptors [40] or the use of SRIF or SRIF receptor agonists, such as the multireceptor ligand pasireotide and the sst2 receptor agonist octreotide [41], are effective in reducing the number of apoptotic neurons, the expression of apoptotic markers, such as caspase-3 mRNA, and the release of glutamate. In contrast, cell death is significantly increased after blocking sst2 receptors with the sst2 receptor antagonist cyanamide [41]. In addition, intravitreal administration of SRIF or sst2 receptor agonists, have been reported to protect the retina from AMPA-induced neurotoxicity *in vivo* [42].

The experimental model that has been used to clarify for the first time the involvement of the sst2 receptor in the SRIF action against retinal ischemia [40] is a retina characterized by over-expression of functional sst2 receptors, caused by the genetic deletion of the sst1 receptor [28, 43-45]. It was expected that sst2 receptor agonist administration to these retinas resulted in increased protection from ischemic damage. However, in contrast to this expectation, a potentiation of the ischemic damage was observed. Indeed, in sst2 receptor over-expressing ischemic retinas, SRIF analogues increased cell death, and octreotide also increased glutamate release. This apparent contradiction has been clarified by experimental data at pharmacological and molecular level showing that over-expressed sst2 receptors are likely to be rapidly desensitized by agonists, thus resulting in a decrease of their functional activity [41]. 

A further effect of sst2 receptor activation in the ischemic retina has been recently reported [46]. The authors reasoned that an ischemic condition not only causes cell death but also induces a vascular response. Therefore, vascular endothelial growth factor (VEGF), one of the main vascular inducers, was investigated in *ex vivo* ischemic mouse retinas under sst2 receptor activation. The results showed a VEGF response to ischemia, in which VEGF is released by retinal neurons suffering ischemic damage and reaches the retinal capillaries, where it is likely to be internalized by endothelial cells. The activation of sst2 receptors has been found to limit VEGF release and the VEGF response. 

Taken together, these observations strongly support an anti-ischemic role of SRIF acting at the sst2 receptor. Less is known of the potential contribution of other SRIF subtype receptors. *In vivo* studies of the rat retina reported no effects of sst1 or sst4 receptor stimulation on neuronal damage induced by AMPA excitotoxicity [42], however a neuroprotective effect in these conditions has been reported upon stimulation of the sst5 receptor [42]. The mechanisms by which the somatostatin analogues prevent the damage produced by chemical ischemia require further elucidation. However, a role of nitric oxide and cyclic GMP has been proposed as mediators of the SRIF protective action against retinal ischemia [47].

### Vasoactive Intestinal Peptide and Pituitary Adenylate Cyclase Activating Peptide

Vasoactive intestinal peptide (VIP) and PACAP are members of the secretin/glucagon/VIP/PACAP/growth-hormone releasing hormone family [48]. VIP is a 28-amino acid peptide, which was first isolated from the gastrointestinal tract [49]. Two PACAP isoforms have been described, one with 27 (PACAP-27) and one with 38 (PACAP-38) amino acids. PACAP-27 is identical to the N-terminal of PACAP-38 [48]. VIP and PACAP receptors belong to the same family of G protein-coupled receptors. Molecular cloning has revealed three distinct PACAP receptor subtypes: a PACAP-specific PAC1 receptor, and two receptors with similar affinity for PACAP and VIP, denominated VPAC1 and VPAC2 receptors, with VPAC2 receptors also displaying high affinity for helodermin. PAC1 receptors are functionally coupled to adenylyl cyclase and to phospholipase C, while VPAC1 and VPAC2 receptors are primarily coupled to adenylyl cyclase. PACAP and VIP have been localized in mammalian retinas [50-55].

PACAP is known to protect the retina against a variety of insults [56]. In particular, a neuroprotective effect of PACAP against retinal ganglion cell loss induced by ischemia following high intraocular pressure in the rat has been reported recently [57]. In addition, ischemic damage is significantly increased in PACAP deficient mice [58]. Moreover, recent observations in an *ex vivo* mouse model of retinal ischemia by adding 10 mM sodium azide to the culture medium show that 1 µM PACAP reduces cell death, glutamate release and oxidative stress (D. Cervia and G. Casini, unpublished data). Since retinal neuronal damage in ischemic conditions is likely to depend, at least in part, by excessive glutamate release, it is interesting to note that PACAP protects against NMDA induced retinal damage [59]. In general, PACAP acts by activating antiapoptotic and inhibiting proapoptotic signaling pathways in the retina [56]. Indeed, the retinoprotective effects of PACAP are not phenotype-specific, but it rather influences general cytoprotective pathways irrespective of the neuronal subtypes in the retina subjected to the effects of ischemia [60]. Finally, recent findings indicate that VIP may also exert neuroprotective effects in the ischemic retina *in vivo*, although it results less effective than PACAP [61].

### Opioid Peptides

The family of opioid peptides is composed of a multiplicity of endogenous opioid ligands that activate G protein-coupled receptors denominated μ, δ, k and ε [62-64]. Enkephalin was detected in inner retinal neurons of guinea pigs [65] and in rat retinal extract [66], while the expression of β-endorphin has been demonstrated in cholinergic amacrine cells of the mouse retina [67]. μ and δ opioid receptors have been detected in rat retina using PCR and Western blot [66], and μ receptors were also detected by immunohistochemistry on processes of bistratified ganglion cells [68]. μ and δ opioid receptors have also been reported recently in ganglion cells as well as in GABAergic and dopaminergic amacrine cells of mouse retinas [69].

Concerning the role of opioid peptides in retinal ischemia, it has been reported that activation of opioid receptors facilitates the development of ischemic preconditioning within the retina and reduces the ischemic damage to the retina [70]. Recent evidence also demonstrates that activation of one or more opioid receptors can reduce the effects of ischemia-reperfusion injury by the suppression of tumor necrosis factor-α production [71]. In addition, intravitreal administration of morphine immediately after reperfusion blunts the effects of ischemia-reperfusion injury, and pharmacologic evidence suggests that this protective action may be mediated, at least in part, by opioid receptors [72-73]. Finally, an involvement of the δ opioid receptor has been reported in mediating the protective effects against ischemic damage of hypoxic preconditioning [66].

### Angiotensin II

Angiotensin is an octapeptide hormone that is known as a potent vasoconstrictor and is part of the renin-angiotensin system. Experimental data have been produced supporting a role for angiotensin II as a neuroactive agent within the CNS [74], including the retina [75]. It mediates its biological actions through the activation of two main receptors: angiotensin type 1 and type 2 receptors. Both receptors have been localized in the rat retina to multiple cell types [76].

There is evidence indicating that ischemia promotes the expression of angiotensin type 1 receptor in the retina and that blocking this receptor may attenuate the retinal damage after ischemia-reperfusion [77]. Similarly, blocking angiotensin type 1 receptor results in protection of retinal neurons in a rat model of glaucoma with chronically elevated intraocular pressure [78]. These protective effects of angiotensin type 1 receptor blockers are associated with reduced glutamate release and reduced oxidative stress provoked by ischemia [77].

### Other Peptides

The roles that SRIF, PACAP, opioid peptides and angiotensin II may play in retinal ischemia are supported by solid experimental evidence documented in several papers. In recent years, however, protective effects against retinal ischemia exerted by other peptidergic substances have also been published in sporadic reports. A study by Szabadfi and coworkers [79] suggests possible protective effects of corticotropin releasing factor (CRF) showing that intraocular administration of urocortin 2, a paralog of CRF that preferentially activates CRF2 receptors, may preserve the thickness of retinal layers and reduce ganglion cell loss in ischemic retinas. In addition, retinal ischemia seems to induce modifications in the retinal endocannabinoid metabolism, and drugs that interfere with the endocannabinoid retinal system may prevent ischemic damage to the retina [80]. Another study reports that NAP, a synthetic 8-amino acid peptide derived from activity-dependent neuroprotective protein, exerts a neuroprotective action *in vivo* after retinal ischemia and optic nerve crush [81]. Furthermore, transgenic mice overexpressing neuroglobin show reduced mitochondrial DNA damage and reduced cell death following ischemia [82], while blocking of the gap junction protein connexin43 using a connexin43 mimetic peptide reduces vascular leakage and ganglion cell death in the ischemic retina [83]. Finally, recent data from our laboratory indicate that substance P, applied at 1 μM concentration to the incubation medium of *ex vivo* mouse retinas with ischemia induced by 10 mM sodium azide, reduces glutamate release, oxidative stress and cell death (D. Cervia and G. Casini, unpublished data).

## CONCLUSION

Despite the rapid development of new pharmacological and surgical modalities, the treatment of retinal disease all too often results in poor final visual acuity. The primary pathologic mechanism underlying suboptimal visual acuity following retinal disease is cell death. The study of neuropeptide systems in the ischemic retina provides a rationale for harnessing the peptidergic receptors as a potential target against retinal neuronal damage. In addition, the data supporting the therapeutic potential of agonists at several of the peptide receptors described in this review continues to increase and these targets clearly merit a thorough evaluation in the clinic. Given the potential advantages of peptides as therapeutic molecules and the technical advances that have occurred in the discovery and development of peptide therapeutics, as for instance new strategies to increase the stability and longevity of peptides *in vivo* and improve their delivery [8], the neuropeptide system might be an exciting new therapeutic option for treating ischemic retinopathies.
